# Neurophysiological Predictors of Response to Medication in Parkinson's Disease

**DOI:** 10.3389/fneur.2021.763911

**Published:** 2021-11-16

**Authors:** Saša R. Filipović, Aleksandra Kačar, Sladjan Milanović, Miloš R. Ljubisavljević

**Affiliations:** ^1^Department for Human Neuroscience, Institute for Medical Research, University of Belgrade, Belgrade, Serbia; ^2^Department of Neurology, Faculty of Medicine, University of Belgrade, Belgrade, Serbia; ^3^Department of Physiology, College of Medicine and Health Sciences, UAE University, Al Ain, United Arab Emirates

**Keywords:** Parkinson's disease, TMS, paired pulse TMS, cortical inhibition, dopaminergic therapy, personalized therapy

## Abstract

**Background:** Although dopaminergic medication has been the foundation of Parkinson's disease (PD) therapy for decades, sensitive and specific therapeutic response biomarkers that allow for better treatment optimization are lacking.

**Objective:** We tested whether the features of Transcranial Magnetic Stimulation-based neurophysiological measures taken off-medication are associated with dopaminergic medication-induced clinical effects.

**Method:** Motor cortex excitability [short-latency intracortical inhibition (SICI), intracortical facilitation (ICF), short-latency afferent inhibition (SAI), and input-output (IO) curve], and plasticity [paired associative stimulation (PAS) protocol] neurophysiological measures were examined in 23 PD patients off-medication. Clinical features were quantified by the motor section of the Unified Parkinson's Disease Scale (total score and lateralized total, bradykinesia, and rigidity sub-scores), and the differences between measures off-medication and on-medication (following the usual morning dose), were determined. Total daily dopaminergic medication dose (expressed as levodopa equivalent daily dose-LEDD), was also determined.

**Results:** SICI significantly correlated with changes in lateralized UPDRS motor and bradykinesia sub-scores, suggesting that patients with stronger basal intracortical inhibition benefit more from dopaminergic treatment than patients with weaker intracortical inhibition. Also, ICF significantly negatively correlated with LEDD, suggesting that patients with stronger intracortical facilitation require less dopaminergic medication to achieve optimal therapeutic benefit. Both associations were independent of disease severity and duration.

**Conclusions:** The results suggest variability of (patho) physiological phenotypes related to intracortical inhibitory and facilitatory mechanisms determining clinical response to dopaminergic medication in PD. Measures of intracortical excitability may help predict patients' response to dopaminergic therapy, thus potentially providing a background for developing personalized therapy in PD.

## Introduction

Although the impairment of afferent dopaminergic innervation from substantia nigra is the core pathophysiological feature common to all patients with Parkinson's disease (PD), while the dopaminergic medication is the best and essentially only available pharmacological treatment, there is a wide variation in symptom progression and severity as well as in response to the treatment (1). There is an increasing interest in subtyping PD and finding reliable biomarkers and predictors of its progression and response to medication (2).

Transcranial magnetic stimulation (TMS), a non-invasive brain stimulation technique, has attracted substantial interest as a probe to investigate brain functioning in health and disease (3). In Parkinson's disease (PD), TMS studies showed various impairments of the primary motor cortex (M1) excitability and plasticity. For example, short interval intracortical inhibition (SICI) is reduced in untreated or 'off-drug' patients (4, 5), and particularly in the most affected patients (6). Similarly, sensorimotor cortical plasticity, tested by paired associative stimulation (PAS), was shown to be diminished in PD patients off medication (7). It is thus tempting to assume that PAS, SICI and other related neurophysiological parameters may be related not only to the presence and severity of the PD, but also can predict the response to medication (7, 8).

Nevertheless, despite some promising results, the robust evidence for measuring cortical excitability and plasticity as biomarkers of disease severity and progression or for predicting the therapeutic response in PD is still limited. Therefore, this study aimed to examine the link between several TMS-based neurophysiological measures and patient's responses to dopaminergic medication. Neurophysiological features of PD patients when off medication were contrasted with medication-induced changes in clinical measures of movement performance.

## Methods

### Patients

Twenty-three people (10 females), diagnosed with idiopathic PD according to the UK Brain Bank criteria (9) participated in the study (Table 1). All participants were on stable, optimized dopaminergic therapy for at least 3 months before entering the study. Only 3 participants were on monotherapy with a dopamine receptor agonist drug, while others had levodopa in their treatment (one was on levodopa monotherapy). For ease of comparison, the daily dopaminergic medication dose was transformed into levodopa equivalent daily dose (LEDD) (10). All participants gave written informed consent; the Ethics Committee of the University of Belgrade Faculty of Medicine approved the study. The study was conducted following the Declaration of Helsinki.

**Table 1:** Clinical characteristics of the studied patients with UPDRS score and lateralized subscores off-medication and on-medication and the (sub) scores' absolute and relative differences.

	**Mean ± SD (Min | Max)**
Age (years)	56.8 ± 8.8 (39 | 72)
Duration of the disease (years)	7.4 ± 5.6 (1 | 20)
Hoehn & Yahr stage	2.6 ± 0.9 (1 | 4)
Levodopa equivalent daily dose^§^ (mg)	768.0 ± 512.7 (75 | 1,750)
UPDRS 3 total score OFF	36.2 ± 14.5 (13 | 68)
UPDRS 3 total score ON	26.8 ± 11.3 (7 | 52)
dUPDRS total score	−9.3 ± 7.6 (-30 |−2) [Z = 4.20 p = 0.00003^*^]
dUPDRS%^†^	−25.2 ± 15.1% (-58.5% |−4.6%)
UPDRS-L subscore–worst side OFF	14.7 ± 4.2 (9 | 26)
UPDRS-L subscore–worst side ON	11.0 ± 4.4 (4 | 19)
dUPDRS-L–worst side	−3.7 ± 2.5 (-10 | 2) [Z = 4.09 p = 0.00004^*^]
dUPDRS-L%–worst side^†^	−26.1% ± 17.5% (-56.0% | 9.7%)
Bradykinesia-L subscore–worst side OFF	9.2 ± 2.6 (6 | 15)
Bradykinesia-L subscore–worst side ON	6.6 ± 2.4 (3 | 11)
dBradykinesia-L–worst side	−2.6 ± 1.8 (-7 | 0) [Z = 4.01 p = 0.00006^*^]
dBradykinesia-L%–worst side^†^	−28.2% ± 16.7% (-54.2% | 0.0%)
Rigidity-L subscore–worst side OFF	9.3 ± 2.6 (6-15)
Rigidity-L subscore–worst side ON	6.8 ± 2.3 (3-11)
dRigidity-L–worst side	−1.0 ± 1.1 (-4 | 1) [Z = 3.39 p = 0.0007^*^]
dRigidity-L%–worst side^†^	−22.0% ± 26.1% (-66.7% | 0.5%)

*UPDRS, Unified Parkinson's Disease Rating Scale [Fahn and Elton, (11)]. OFF, off-medication; ON, on-medication; L, lateralized*.

§ *Levodopa equivalent daily dose (LEDD) calculated according to the conversion coefficients suggested by Tomlinson et al. (10)*.

† *Relative on-medication–off-medication difference expressed as ratio between the difference and the off-medication value*.

* *Wilcoxon Matched Pairs Test*.

### Study Design

Participants were assessed twice: once off-medication, after an overnight (i.e., more than 12 h) withdrawal, and once on-medication, following the usual morning dose, in their subjectively defined optimal ON state. Clinical measures were taken in both off-medication and on-medication states, while neurophysiological measures were taken only in the off-medication state. Clinical assessor was blinded for the patient's medication status. The assessments were carried out at the same time of the day to avoid diurnal fluctuations. The order of the off-medication and the on-medication assessments was counterbalanced across participants.

### Clinical Measures

Participant's motor state was assessed using the 3rd (motor) part of the Unified Parkinson's Disease Rating Scale (UPDRS) (11). In addition to the total UPDRS motor score, a set of lateralized sub-scores from the limbs of the more affected side were calculated: lateralized UPDRS motor sub-score (the sum of scores on items 20–26), lateralized Bradykinesia sub-score (the sum of scores on items 23–26), and lateralized Rigidity sub-score (sum of scores on item 22).

To assess the differences in the motor state (total UPDRS motor score and lateralized UPDRS motor, lateralized Bradykinesia, and lateralized Rigidity sub-scores) between off-medication and on-medication states (i.e., the dopaminergic therapy effect) directly measured values were used. To compensate for the differences in absolute numbers' ranges between total UPDRS motor score and derived lateralized sub-scores, for assessment of the correlations between the effect of dopaminergic therapy and neurophysiological measures, normalized relative score differences were calculated (*dUPDRS%, dUPDRS-L%, dBradykinesia-L%*, and *dRigidity-L%*, respectively), using the formula: *(ON score – OFF score)/OFF score* (expressed in percentages); negative values signified an improvement.

### Neurophysiological Measures

For neurophysiological recordings, participants were seated comfortably with arms supported by adjustable armrests. All neurophysiology measurements were carried out on the clinically more affected side. Surface EMG was recorded from the abductor pollicis brevis (APB) muscle of the target arm using 0.9 cm Ag–AgCl electrodes placed in a belly-tendon montage. The EMG signals were amplified (1000x), and filtered (10–2000 Hz), using the DAM 50 differential amplifier (World Precision Instruments, USA). The data was digitized online (4 kHz/channel) via the “CED 1401 plus” interface (Cambridge Electronics Design, UK), and stored on the computer. The EMG signals were displayed on an oscilloscope to provide subjects with feedback. All neurophysiological measures were taken with the muscle at rest.

TMS was applied using a figure-of-eight coil (outer coil diameter 70 mm), with two Magstim 200 magnetic stimulators connected with a Magstim Bistim unit (Magstim, UK). The coil was held tangentially to the skull with the handle pointing backwards and laterally at an angle of 45° to the sagittal plane. The coil was positioned at the optimal scalp position for eliciting a motor evoked potential (MEP). Peak-to-peak MEP amplitudes were determined in all neurophysiological measures.

First, to allow further measurements, the rest motor threshold (RMT) was determined as the minimum stimulus intensity (expressed as a percentage of maximum stimulator output), required to produce a MEP of at least 50 μV in the relaxed muscle in 3 out of 5 consecutive trials.

Paired-pulse TMS protocol consisting of a sub-threshold (90% RMT) conditioning stimulus followed by a supra-threshold (120% RMT) test stimulus, delivered either after 3 ms or 10 ms, was used for the *short-latency intracortical inhibition (SICI)* and the *intracortical facilitation (ICF)* measurements, respectively (12). SICI and ICF trials were randomly intermixed with trials consisting of unconditioned test stimulus only and delivered with an intertrial interval of 5 ± 1 s; fifteen of each of the three trial types were delivered, 45 in total. The SICI and ICF values were expressed as ratios, *conditioned MEP/unconditioned MEP* (in percentages). For SICI, smaller values correspond with higher inhibition; larger values corresponded with higher facilitation for ICF.

To assess the *input-output (IO) curve*, MEPs were recorded with TMS intensities of 100, 110, 120, 130, and 150% of RMT; eight trials for each of the intensities. The order of presentation of the conditions was pseudo-random, and stimuli were delivered every 5 s. The average MEP amplitudes for each intensity were plotted, and the area under the curve (*AUC-IO*) was calculated as a surrogate marker of the overall corticospinal output (13).

To investigate *plasticity*, a relative increase in MEP size following paired associative stimulation (PAS) protocol was examined. In the PAS protocol, 180 pairs of conditioning-test stimuli, with 25 ms inter-stimulus interval, were delivered at a 0.2 Hz rate. The conditioning stimulus was percutaneous electrical square wave pulse (constant current; pulse width of 200 μs, intensity 300% of perceptual threshold) delivered to the median nerve at the wrist, while the test stimulus was TMS pulse (120% RMT) delivered over the contralateral motor cortex (14, 15). Ten trials of test stimuli, with an intertrial interval of 5 ± 1 s, were recorded immediately after the PAS protocol (*PAS MEP1*) and 30 min later (*PAS MEP2*). The mean amplitudes for each time point were calculated as well as the mean of the ten trials recorded before the PAS (baseline). The values for both measures were expressed as ratios: *PAS MEP/baseline MEP* (in percentages).

In addition, to measure *short-latency afferent inhibition (SAI)*, following the procedure described by Stefan et al. (14), we used the first 20 conditioned MEPs from the PAS protocol. The SAI was expressed as a ratio (in percentages) of the average of the conditioned MEPs to the baseline MEP; smaller values corresponded to stronger inhibition.

### Data Analysis

Given the non-interval nature of the UPDRS score and derived sub-scores, all analyzes were carried out with non-parametric statistic-Wilcoxon Matched Pairs Test, for differences in the clinical features between off-medication and on-medication states, and Spearman rank-order correlation test for correlations between the effect of dopaminergic therapy and neurophysiological measures. The level of significance was set at *P* < 0.05.

## Results

Table 1 summarizes clinical measures off and on medication as well as their absolute and relative differences. As expected, the total UPDRS motor score and all lateralized sub-scores were significantly higher off-medication than on-medication. The average relative difference was about 25% across the clinical (sub) scores. However, none of the relative differences between off-medication and on-medication (sub) scores correlated with the age, duration, Hoehn and Yahr stage, and total off-medication UPDRS motor score (|*R*| < 0.38, *p* > 0.07 in all cases). The only exception was the relative difference in lateralized rigidity sub-score (dRigidity-L%) which showed a negative correlation with duration of the disease and off-medication UPDRS motor score (*R* = −0.46, *p* = 0.028 and *R* = −0.52, *p* = 0.011, respectively). A more negative sub-score difference (i.e., larger improvement) was associated with longer duration and higher total UPDRS score.

The total daily dose of dopaminergic medication required to achieve optimal clinical benefit (expressed as levodopa equivalent daily dose–LEDD), correlated with duration of the disease, Hoehn and Yahr stage, and total UPDRS motor score (*R* = 0.74, *p* = 0.00005; *R* = 0.55, *p* = 0.006; and *R* = 0.58, *p* = 0.004, respectively); correlation with age was at the edge of significance (*R* = 0.40, *p* = 0.057). The total daily LEDD did not correlate with any of the relative differences between off-medication and on-medication (sub) scores (|*R*| < 0.12, *P* > 0.59 in all cases); only dRigidity-L% showed a negative correlation with LEDD (*R* = −0.42, *p* = 0.045) – larger medication-induced improvement was associated with higher LEDD.

**Figure 1:**
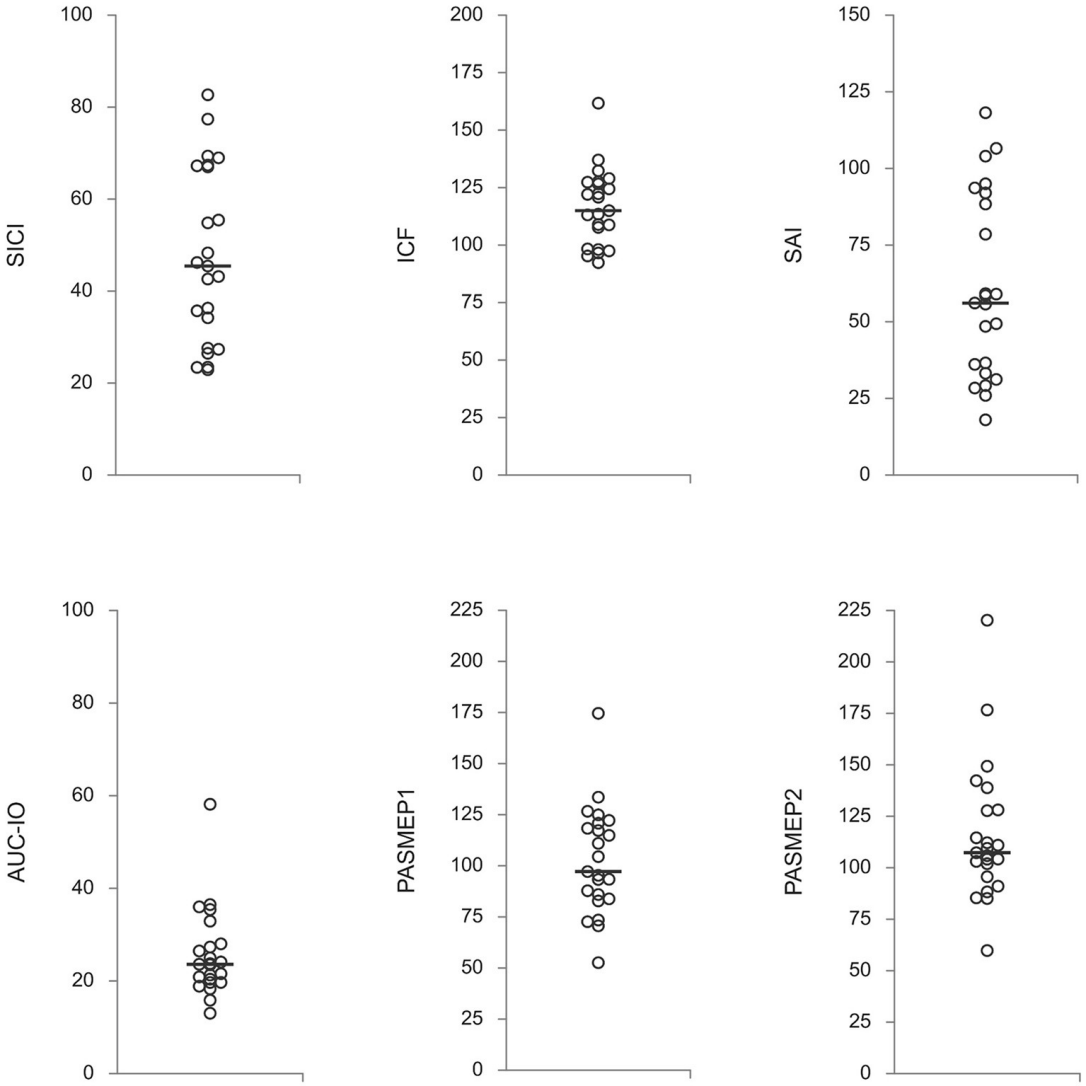
Neurophysiological measures off-medication. Presented are individual subjects' values with a median (horizontal line) for each measure. SICI, short-latency intracortical inhibition; ICF, intracortical facilitation; SAI, short-latency afferent inhibition; AUC-IO, area under the input-output curve; PASMEP1 and PASMEP2–motor evoked potentials (MEP) measured immediately and 30 min after, respectively, paired associative stimulation protocol. SICI, ICF, and SAI are presented as ratios *conditioned-MEP/test-MEP* (in percentages). AUC-IO is presented as *mVx%RMT* (%RMT–resting threshold in percentages of the maximal stimulator output). PASMEP1 and PASMEP2 are presented as ratios *post-PAS MEP/before-PAS MEP* (in percentages).

**Figure 2:**
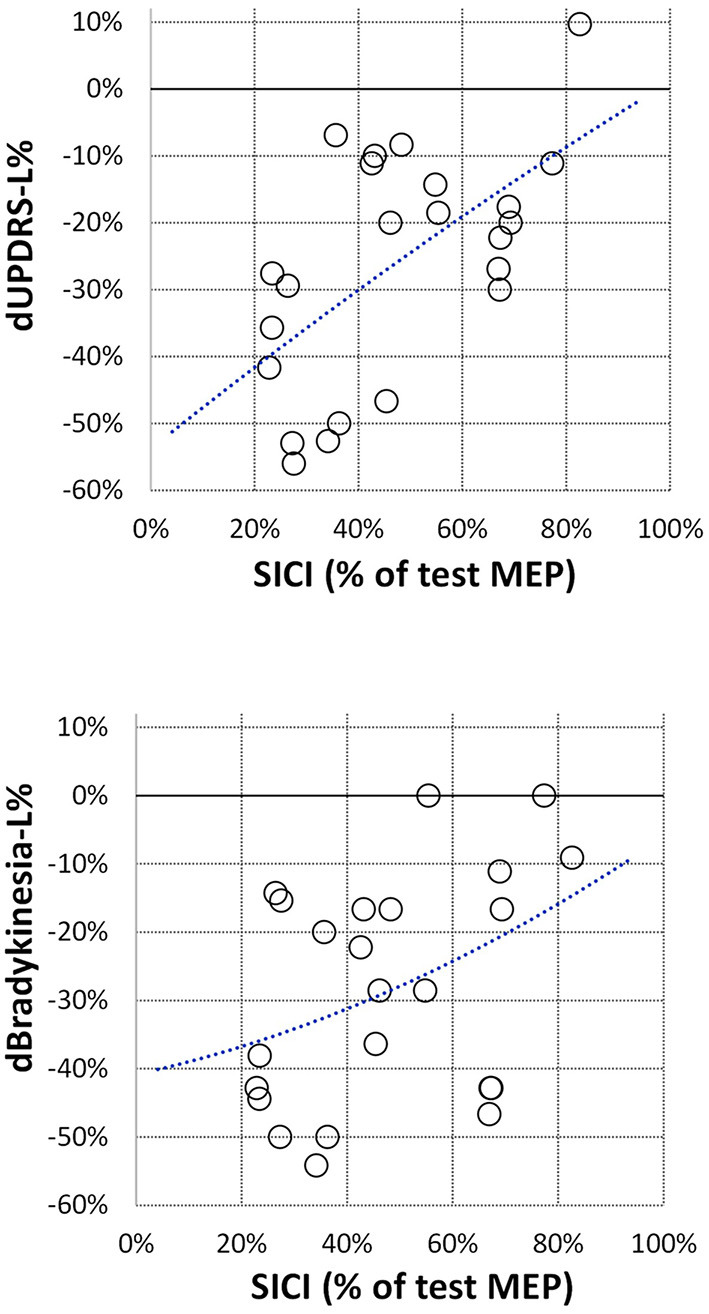
Correlation between off-medication SICI and relative changes in lateralized UPDRS sub-score (dUPDRS-L%) and in lateralized bradykinesia sub-score (dBradykinesia-L%). SICI is presented the same as in Figure 1; relative changes in clinical sub-scores are presented as ratios *(on-medication–off-medication)/off-medication*. The polynomial regression lines are plotted on each graph for illustrative purposes only.

Off-medication neurophysiological measures are presented in Figure 1. They did not correlate with patients' age, disease duration, Hoehn & Yahr stage, and total UPDRS motor score (|*R*| < 0.39, *p* > 0.06 in all cases). Correlations between off-medication neurophysiological measures and medication-induced changes in motor scores are presented in Table 2. Only two correlations were found to be significant, and both involved SICI. The off-medication SICI showed positive correlations with the effects of dopaminergic treatment on lateralized UPDRS motor sub-score (dUPDRS-L%) and lateralized bradykinesia sub-score (dBradykinesia-L%) on the more affected side (Figure 2). The better the SICI (i.e., the stronger intracortical inhibition manifested by the lower SICI-conditioned MEP), the larger was the relative medication-induced improvement in the lateralized UPDRS and bradykinesia sub-scores.

**Table 2:** Correlations between neurophysiological measures and medication induced relative changes in clinical measures.

	**dUPDRS% total score**	**dUPDRS-L% worst side**	**dBradykinesia-L% worst side**	**dRigidity-L% worst side**	**LEDD**
SICI	0.26 (*p* = 0.23)	**0.54 (*****p*** **= 0.007)**	**0.44 (*****p*** **= 0.036)**	0.20 (*p* = 0.40)	−0.17 (*p* = 0.43)
ICF	0.02 (*p* = 0.93)	−0.02 (*p* = 0.94)	0.22 (*p* = 0.32)	0.29 (*p* = 0.23)	**-0.55 (*****p*** **= 0.006)**
SAI	0.36 (*p* = 0.10)	0.33 (*p* = 0.12)	0.32 (*p* = 0.14)	0.07 (*p* = 0.77)	−024 (*p* = 0.28)
AUC-IO	0.38 (*p* = 0.07)	0.10 (*p* = 0.65)	0.19 (*p* = 0.39)	0.02 (*p* = 0.93)	−0.12 (*p* = 0.60)
PASMEP1	−0.14 (*p* = 0.51)	−0.12 (*p* = 0.58)	−0.28 (*p* = 0.20)	−0.34 (*p* = 0.16)	−0.08 (*p* = 0.73)
PASMEP2	−0.04 (*p* = 0.84)	0.23 (*p* = 0.29)	0.27 (*p* = 0.22)	0.15 (*p* = 0.55)	−0.13 (*p* = 0.55)

*Presented are values of Spearman R with probability of null hypothesis in brackets. Significant results are marked in bold. UPDRS, Unified Parkinson's Disease Rating Scale [Fahn and Elton, (11)]; d%, relative difference; L, lateralized; SICI, short-latency intracortical inhibition; ICF, intracortical facilitation; SAI, short-latency afferent inhibition; AUC-IO, area under the input-output curve; PASMEP1 and PASMEP2, motor evoked potentials (MEP) measured immediately and 30 min after, respectively, paired associative stimulation protocol (PAS)*.

**Figure 3:**
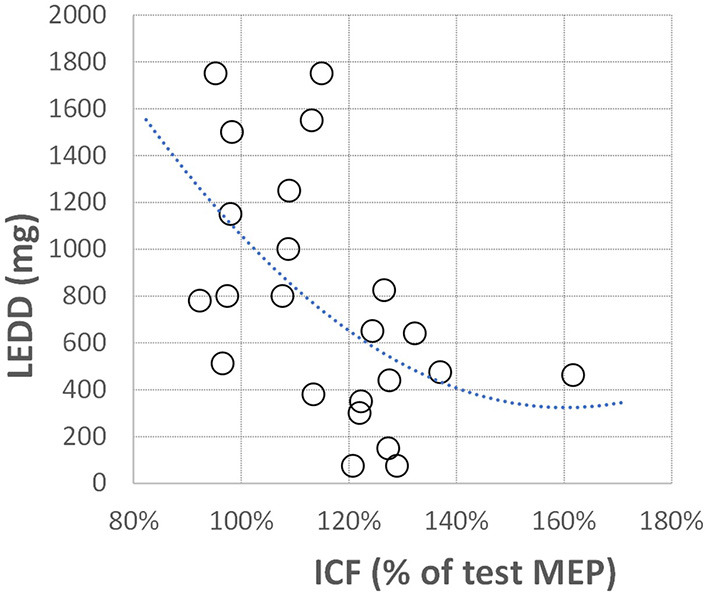
Correlation between off-medication ICF and total daily dopaminergic medication dose expressed as levodopa equivalent daily dose (LEDD). ICF is presented the same as in Figure 1; LEDD is presented in milligrams. The polynomial regression line is plotted for illustrative purposes only.

In addition, we looked at the possible correlation between the total daily dopaminergic medication dose (as LEDD) and neurophysiological measures (Table 2). Only one correlation was found to be significant. ICF from the more affected hand showed a strong negative correlation with the daily dopaminergic drug dose (Figure 3). The better the ICF (i.e., the stronger intracortical facilitation manifested by the larger ICF-conditioned MEP), the lesser the total daily dose of dopaminergic medication was required to achieve optimal clinical benefit. There was no correlation between SICI and ICF values in our patients (*R* = 0.07, *p* = 0.74).

## Discussion

This study examined whether neurophysiological measures of motor cortex excitability and plasticity in an off-medication state could predict clinical response to dopaminergic therapy. To secure a large enough variability of the clinical measures allowing for potential correlations to be detected, we recruited PD patients with a wide range of features such as age, disease duration, severity, and amount of regular dopaminergic medication providing optimal therapeutic benefit. In keeping with their clinical differences, the patient's clinical responses (motor part of the UPDRS) measured by the change in the scale's scores between off-medication and on-medication states, varied widely–from only 5% to almost 60% of the off-medication state. The minimal medication-induced change of our sample's total motor UPDRS score was 2 points corresponding to the so-called Minimal Clinically Important Change in the UPDRS motor score (16, 17). Likewise, the lateralized motor UPDRS sub-score and the more detailed derived sub-scores for bradykinesia and rigidity also showed clear medication-induced improvement.

The impairments in the neurophysiological measures of motor cortex excitability and plasticity in PD are well established [e.g., (7, 18)]. In this study, we collected a set of measures that included intracortical facilitation and inhibition (ICF and SICI, respectively), corticospinal excitability (IO curve), sensory-motor interaction and inhibition (SAI), and plasticity (PASMEP1 and PASMEP2). All measures were taken from the more affected side with patients in an off-medication state.

### Short-Term Intracortical Inhibition

Short interval intracortical inhibition (SICI), elicited by applying a conditioning stimulus 1–6 ms before the test stimulus (12), is a common measure of cortical inhibitory mechanisms. There is robust supporting evidence demonstrating the cortical origin of SICI (19) related to GABA_A_ mediated cortical inhibition (20, 21).

The main result of our study is that, in patients with Parkinson's disease, a stronger intracortical inhibition off-medication is associated with a better improvement of motor functions on dopaminergic therapy, i.e., patients whose intracortical inhibition is better preserved seem to benefit more from dopaminergic treatment.

Most of the studies investigating SICI found it to be on average lower (i.e., lesser suppression of the test MEP amplitude) in PD patients off-medication than in controls, suggesting the presence of impaired intracortical inhibition in PD (4, 5, 22–27). In the most recent study, with the largest number of participants so far, Ammann et al. (28) confirmed this finding. They also confirmed our previous finding (25) that impaired SICI is a ubiquitous finding in PD patients, regardless of the severity of the disease and previous exposure to dopaminergic treatment (i.e., whether patients were dopaminergic drugs naïve or were chronically treated). In keeping with other similar studies, our study's individual patients' SICI values varied widely, from very strong inhibition to almost no inhibition. However, they were not related to any clinical feature (i.e., patients age, disease duration, severity and stage of the disease, and total daily dopaminergic drug dose). Most of the other studies found the same [e.g., (24, 25, 28)]. Moreover, dopaminergic medication was not shown to be able to improve the impaired SICI consistently, despite clear improvement of clinical features (24, 26, 27). Therefore, it can be concluded that SICI does not directly contribute to the pathophysiology of motor symptoms in PD. Instead, it seems to be impaired from the onset of the disease and appears not to be affected much by further disease progression and pharmacological interventions. Large variability of SICI in PD recorded across various studies, including our own, together with lack of correlation with disease duration, could be thus taken as an indicator for the presence of various physiological phenotypes of PD with various levels of intracortical inhibition impairment as a constant trait. The link between preserved SICI and a better clinical response to dopaminergic treatment may be interpreted along the same lines. It seems as if preserved intracortical inhibition is necessary for dopaminergic medication to exert its functional effects on improving clinical symptoms. The finding that the *absolute* dose of medication, expressed in LEDD, required to achieve the optimal clinical benefit did not correlate with the off-medication SICI could further support this hypothesis. It appears as if it is not the absolute dose of medication, but the preservation of the integrity of the intracortical inhibitory mechanisms that is important for the optimal dopaminergic medication therapeutic benefit. The presence of a significant correlation between dopaminergic medication dose required for optimal therapeutic benefit and disease duration, Hoehn and Yahr stage, and severity of motor symptoms lends further support to this assumption.

However, whether this variability in individual SICI values seen in PD patients reflects the variability of the measure in a healthy population (29, 30) or is a result of the variable level of an initial hypothetical injury very early in the disease process is an open question. Whatever is the case, it looks from our results that defective intracortical inhibition is a strong predictor of, but quite likely also a risk factor for, worse clinical response to dopaminergic medication in PD. It is tempting to wonder whether co-administration of a GABAa agonist and dopaminergic drugs could boost the clinical response in patients with low SICI. Further research on larger samples of patients could shed more light on this issue.

Although off-medication SICI correlated with medication-induced improvement in lateralized total UPDRS motor sub-score of the limbs on the same side, on detailed assessment it turned out that SICI correlated with the same side medication-induced improvement in bradykinesia, but not with the improvement of rigidity. This could point toward the specificity of the link between the two measures, the SICI and the medication-induced bradykinesia improvement. In the UPDRS motor part, bradykinesia is tested by repetitive movements requiring rapid alteration between agonists and antagonists' activity. For these movements to be appropriately accomplished, both rapid activation and rapid deactivation (i.e., inhibition) of the target muscles are equally important. It may be assumed that while dopaminergic medication boosting the activity of the facilitatory motor cortex mechanisms helps improve the activation segment, the level of preserved intracortical inhibition determines the effectiveness of the deactivation segment of muscle contractions, thus affecting the successfulness of the attempted movements. Impaired scaling of the agonist and antagonist bursting patterns, which is variably and incompletely improved by dopaminergic medication, is a known feature of PD (31, 32).

In contrast, the medication-induced change in another cardinal PD symptom, rigidity, did not correlate with either SICI or any other tested neurophysiologic measure. This was in stark contrast with bradykinesia, suggesting their different pathophysiology. The absence of correlation between neurophysiological measures and medication-induced change in rigidity is unclear as the underlying mechanisms of rigidity are poorly understood (33). Moreover, as contributions from the spinal cord, brain stem and higher cortical circuits have all been proposed (34) it may be that the used neurophysiological measures were not sensitive enough to capture specific changes related to rigidity.

### Intracortical Facilitation

Another significant result of our study was a negative correlation between ICF (i.e., increase of the test MEP amplitude), and the total daily dose of dopaminergic medication required to achieve optimal therapeutic benefit. In other words, the stronger off-medication ICF, the lesser dose of dopaminergic drugs was needed.

Intracortical facilitation (ICF) is a common TMS measure of cortical facilitatory mechanisms. It involves applying a conditioning stimulus 8–30 ms before the test stimuli (12). ICF appears to be mediated by neuronal population separate from SICI (35, 36). The ICF in PD has been consistently found not to differ from controls (23, 25, 26, 37). Dopaminergic medication was not found to affect ICF in PD (26, 28). We are not aware of other studies investigating the relationship between off-medication ICF and dopaminergic medication in a way that could be directly comparable with our study. The ICF did not correlate with any clinical features (including duration and severity of disease), or patients' demographics. A similar finding was reported in some of the previous studies [e.g., (25, 26)]. This would suggest that intracortical facilitatory mechanisms, as measured by ICF, are not primarily impaired in PD. Instead, it seems that the subcortical drive necessary to “feed” facilitation gets progressively weaker, requiring a higher dose of dopaminergic medication to keep it working. In support of this hypothesis, in our study, the dopaminergic medication dose required for optimal therapeutic benefit correlated highly significantly with disease duration, Hoehn and Yahr stage, and severity of motor symptoms. Unfortunately, we are not aware of any other study that looked at similar interactions allowing the comparison with the current findings.

### Other Measures of Cortical Excitability (Input-Output Curve and Short-Latency Afferent Inhibition)

The input-output (IO) curve has been rarely investigated in PD. The scarce studies reported diverse findings in patients off-medication, from diminished (25) over normal (23) to increased (26) IO curve slope. The AUC-IO measured in an off-medication state in our study did not correlate with medication-induced improvement in any of the clinical measures. The off-medication AUC-IO did not correlate with any of the clinical and demographic features either. We are not aware of any other study examining the predictive value of the off-medication IO curve regarding medication-induced clinical improvement. However, Bologna et al. (26) did not find a correlation between medication-induced improvement in IO curve slope and improvement in clinical measures.

Application of conditioning stimuli to a peripheral nerve followed by a test stimulus over the contralateral motor cortex 2–8 ms after the arrival of the afferent volley to the somatosensory cortex causes a reduction of MEP, a phenomenon termed short-latency afferent inhibition (SAI) (38). Short-latency afferent inhibition (SAI) has attracted considerable attention in PD research (39). However, only a few studies included patients off-medication, and they generally reported no difference between patients and healthy controls [e.g., (40, 41)]. Same as Sailer et al. (40) we did not find that off-medication SAI correlated with clinical and demographic features, which, together with its essentially normal values, could explain the lack of correlation between SAI values and medication-induced clinical improvement in our study.

### Paired-Associative Stimulation Induced Plasticity

In PD patients, PAS-related cortical plasticity was found to be impaired off-medication and restored by dopaminergic medications in non-dyskinetic but not in the dyskinetic PD patients (26, 42, 43). Impaired PAS in PD patients suggests deficient LTP-like effects in the motor cortex of PD patients. One possible explanation could be that the dopaminergic deficiency may prevent the motor cortex from changing the synaptic connection strength when primed by a repetitive, low-frequency stimulation (43). We did not find an association between PAS-induced motor cortex plasticity off-medication and medication-induced changes in any of the measures of motor performance. It may be thus concluded that the capacity for dopaminergic medication induced clinical improvement does not depend on the level of the preserved motor cortex plasticity. This may be due to the converging evidence from animal and human studies that cortico-striatal plasticity in PD is modulated not only by the dopaminergic loss, but also by changes in other neurotransmitter systems, such as acetylcholine, nitric oxide, and endocannabinoids (44).

### General Remarks

Bologna et al. (26) also examined the association between M1 excitability and plasticity measures and movement performance in both off and on dopaminergic medication. They showed that administration of dopaminergic therapy, besides improvements in motor performance, improved M1 excitability and plasticity (i.e., the slope of input-output MEP curve and PAS plasticity) in comparison to the off-medication state. However, they did not find any correlation between dopaminergic medication-induced changes in neurophysiological and clinical measures. Unfortunately, neither this nor any other study examined the relationship between off-medication neurophysiological measures and changes in movement characteristics induced by dopaminergic medication, thus preventing direct comparison with the results of this study.

There are some potential limitations of this study. First, the study was performed on relatively small sample size, potentially affecting the statistical analysis, which could have been underpowered. Secondly, although we tried to include an extensive sample encompassing wide clinical features, we cannot rule out that the relationship we found (between off-medication SICI and motor response to dopaminergic medication) could be due to a common correlation with disease progression and associated changes in M1 excitability (45). However, this seems unlikely since there was no correlation between neurophysiological data and off-medication clinical features. Moreover, although the neurophysiological measures were taken in an off-medication state, residual effects of chronic exposure to dopaminergic medication could not be entirely ruled out. However, we do not believe that this factor has affected the results since no correlation between neurophysiological measures and duration and severity of the disease, and total daily dopaminergic medication dose was found.

Another potential study limitation may be related to the use of the UPDRS scale (11) instead of the MDS-UPDRS version (46) which could not be used due to the lack of an official Serbian translation. Nevertheless, a high correlation between the two versions of the scale for the part 3 (motor examination) scores was established already when the MDS-UPDRS version was presented (46) and was confirmed in subsequent studies [e.g., (47–49)]. Moreover, since in this study we were not interested in absolute score values but in the relative change in the scores induced by medication, and since the two versions were shown to be equally sensitive to the medication-induced change (48), we believe that the use the UPDRS scale did not impact the study outcomes.

Finally, it should be kept in mind that various sources of variability may affect TMS-derived neurophysiological measures of cortical excitability and plasticity, even in healthy subjects (29, 30). Longitudinal studies with a more significant number of patients in different stages of the disease are needed to establish whether preservation of intracortical inhibitory and facilitatory circuits is directly related to the effectiveness of dopaminergic medication and the consequent motor improvement.

## Conclusions

Although dopaminergic therapy has been the foundation of Parkinson's disease therapy for decades, there is high individual variability in treatment response. Furthermore, it is well recognized that the effects of dopaminergic therapy deteriorate after years of treatment. Thus, predicting optimal treatment response based on validated specific and sensitive biomarkers is needed to facilitate personalized treatments in PD. This study presented evidence suggesting that preservation of functional integrity of the intracortical inhibitory and facilitatory mechanisms renders better motor clinical response to dopaminergic therapy in PD. This is a novel finding for which we have not been able to find an analogous literature report. If confirmed in a larger sample(s) of patients with PD, it will result in a better understanding of the physiological background of individual differences in response to dopaminergic treatment. The finding may also be a potential biomarker in delivering better prediction of patients' response to dopaminergic medication.

## Data Availability Statement

The raw data supporting the conclusions of this article will be made available by the authors, uppon reasonable request.

## Ethics Statement

The studies involving human participants were reviewed and approved by the Ethics Committee of the University of Belgrade Faculty of Medicine. The patients/participants provided their written informed consent to participate in this study.

## Author Contributions

SF was involved in the conceptualization and design of the study, data collection, statistical analysis, organization and presentation of the data, writing, review, and critique of the manuscript. AK was involved in the organization and execution of the study, data collection, and critique of the manuscript. SM was involved in analyzing data and critique of the manuscript. ML was involved in the design of the study, writing of the first draft, and review and critique of the manuscript. All authors have read and agreed to the published version of the manuscript.

## Funding

This study was supported by Project Grants (#175012 and #175090) from the Ministry for Education, Science, and Technological Development of the Republic of Serbia. SF and SM were partially supported by institutional support to the Institute for Medical Research by the Ministry of Education, Science and Technological Development of the Republic of Serbia (contract: 451-03-9/2021-14/200015). ML was partially supported by Aspire grant AARE19-060.

## Conflict of Interest

The authors declare that the research was conducted in the absence of any commercial or financial relationships that could be construed as a potential conflict of interest.

## Publisher's Note

All claims expressed in this article are solely those of the authors and do not necessarily represent those of their affiliated organizations, or those of the publisher, the editors and the reviewers. Any product that may be evaluated in this article, or claim that may be made by its manufacturer, is not guaranteed or endorsed by the publisher.
